# Synthesis, crystal structure and Hirshfeld surface analysis of di­aqua­bis­(*o*-phenyl­enedi­amine-κ^2^ *N*,*N*′)nickel(II) naphthalene-1,5-di­sulfonate

**DOI:** 10.1107/S2056989023009350

**Published:** 2023-10-26

**Authors:** Jabbor R Suyunov, Khayit Kh. Turaev, Bekmurod Kh. Alimnazarov, Yusuf E. Nazarov, Islombek J. Mengnorov, Bakhtiyar T. Ibragimov, Jamshid M. Ashurov

**Affiliations:** a Termez State University, "Barkamol avlod", at street, 43., Termez city, Uzbekistan; b Institute of Bioorganic Chemistry, Academy of Sciences of Uzbekistan, 100125, M. Ulugbek Str 83, Tashkent, Uzbekistan; Vienna University of Technology, Austria

**Keywords:** *o*-Phenyl­enedi­amine, 1,5-naphthalene­disulfonic acid, crystal structure, inter­molecular inter­actions, hydrogen bonding, Hirshfeld surface

## Abstract

The reaction of *o*-phenyl­enedi­amine (OPD), sodium naphthalene1,5-di­sulfonate (Na_2_NDS) and nickel sulfate in an ethanol–water mixture yielded the title compound, [Ni(OPD)_2_(H_2_O)_2_]·NDS or [Ni(C_6_H_8_N_2_)_2_(H_2_O)_2_](C_10_H_6_O_6_S_2_). This salt consists of a complex [Ni(OPD)_2_(H_2_O)_2_]^2+^ cation with two bidentate OPD ligands and *trans* aqua ligands, and a non-coordinating NDS^2–^ anion, which is the double-deprotonated form of H_2_NDS. The Ni^II^ atom is situated at a center of inversion and exhibits a slightly tetra­gonally distorted {O_2_N_4_} octa­hedral coordination environment, with four shorter equatorial Ni—N bonds [2.0775 (17) and 2.0924 (18) Å] and a longer axial Ni—O bond [2.1381 (17) Å]. The OPD ligand is located about an inversion center and is nearly coplanar with the NiN_4_ plane [dihedral angle 0.95 (9)°]. In the crystal, the cations and anions are connected by charge-assisted inter­molecular N—H⋯O and O—H⋯O hydrogen-bonding inter­actions into the tri-periodic network structure. Hirshfeld surface analysis indicates that the most important contributions to the crystal packing are from H⋯H (44.1%), O⋯H/H⋯O (34.3%), C⋯H/H⋯C (14.8%) C⋯C (6.5%) (involving the cations) and O⋯H/H⋯O (50%), H⋯H (25%), C⋯H/H⋯C (15.3%), C⋯C (8.2%) (involving the anions) inter­actions.

## Abstract 2

In the title salt, [Ni(C_6_H_8_N_2_)_2_(H_2_O)_2_]·(C_10_H_6_O_6_S_2_), the Ni^II^ atom exhibits a slightly tetra­gonally distorted {O_2_N_4_} octa­hedral coordination environment, with two pairs of equatorial Ni—N bonds and a pair of longer axial Ni—O bonds.

## 1. Chemical context

*o*-Phenyl­enedi­amine (OPD) condenses with ketones and aldehydes to a variety of useful products. Its reactions with carb­oxy­lic acids and their derivatives produce the important class of benzimidazoles (Vishvanath & Ketan, 2014 ▸; Aniket *et al.*, 2015 ▸; Pardeshi & Thore, 2015 ▸). Hence, OPD is commonly used in various industrial processes, including the production of dyes, polymers and the synthesis of fungicides, corrosion inhibitors, pigments, and pharmaceuticals (Abdullah *et al.*, 2019 ▸; Sagasser *et al.*, 2019 ▸; Pisarevskaya *et al.*, 2020 ▸; Jadoun *et al.*, 2021 ▸). It also exhibits electrical conductivity and is used in the production of conductive materials, such as sensors and batteries (Sayyah *et al.*, 2009 ▸; Bottari *et al.*, 2020 ▸). OPD is also a versatile ligand in coordination chemistry. It forms complexes with different metal ions, such as lanthanides (Koroteev *et al.*, 2020 ▸), zinc (González Guillen *et al.*, 2018 ▸; Zick & Geiger, 2016 ▸), cobalt (Ngopoh *et al.*, 2015 ▸; Konieczny *et al.*, 2019 ▸), copper (Djebli *et al.*, 2012 ▸; Chakraborty *et al.*, 2014 ▸), cadmium (González Guillen *et al.*, 2018 ▸) or nickel (Sabbani & Das, 2009 ▸; Lu *et al.*, 2009 ▸; Willett *et al.*, 2012 ▸; Adhikari *et al.*, 2021 ▸).

Compounds comprising 1,5-naphthalene­disulfonic acid (H_2_NDS) or its deprotonated form (sulfonates) are of inter­est in supra­molecular chemistry (Shi *et al.*, 2014 ▸; Xu *et al.*, 2019 ▸; Chen *et al.*, 2020 ▸), because the sulfonate group can accept up to six hydrogen bonds (Chen *et al.*, 2020 ▸; Oh *et al.*, 2020 ▸; Chen *et al.*, 2022 ▸). H_2_NDS can react with organic compounds under formation of organic cations and the NDS^2–^ anion, or with metal compounds either under formation of non-coordinating NDS^2–^ anions, or with NDS^2–^ as a ligand (Huo *et al.*, 2005 ▸; Kokunov *et al.*, 2015 ▸). As a ligand, NDS^2–^ can bind in a bridging mode (Lian & Qu, 2013 ▸; Das *et al.*, 2015 ▸; Tai *et al.*, 2015 ▸). 
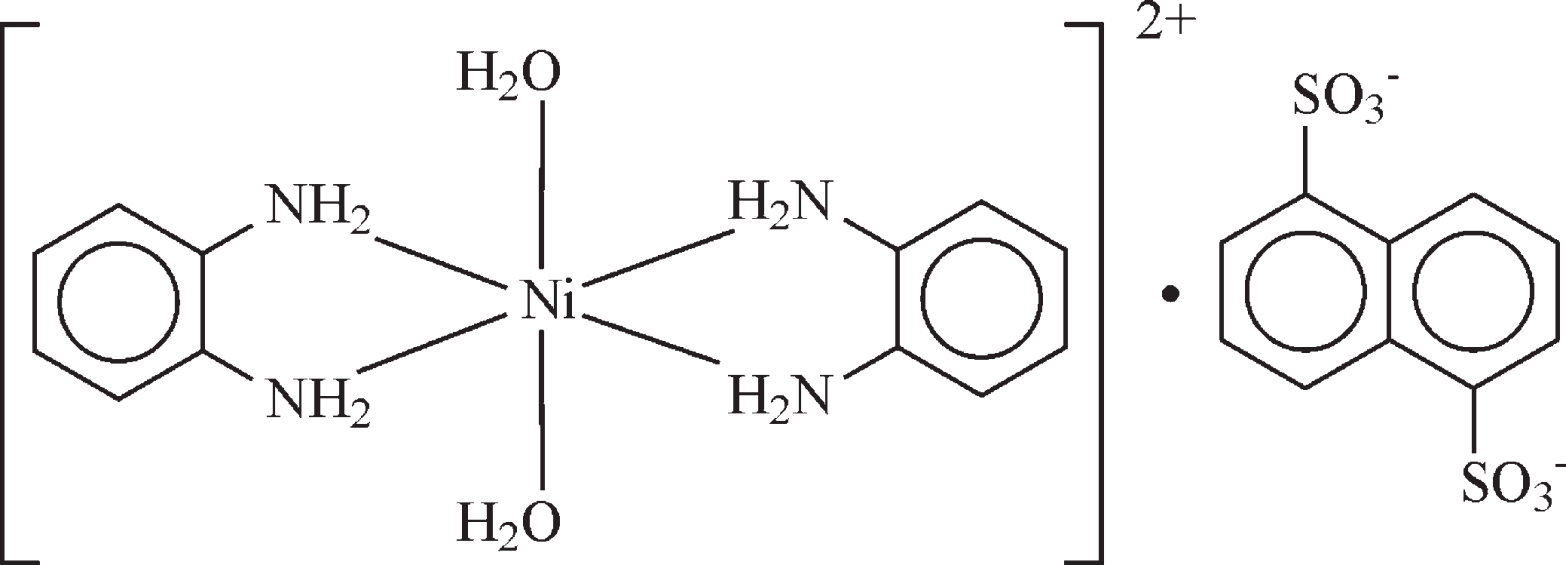


In this work, we focus on the synthesis, crystal structure, and Hirshfeld surface analysis of a nickel(II) complex, [Ni(OPD)_2_(H_2_O)_2_]·NDS, where the NDS^2–^ anion is not part of the metal coordination sphere.

## 2. Structural commentary

**Figure 1:**
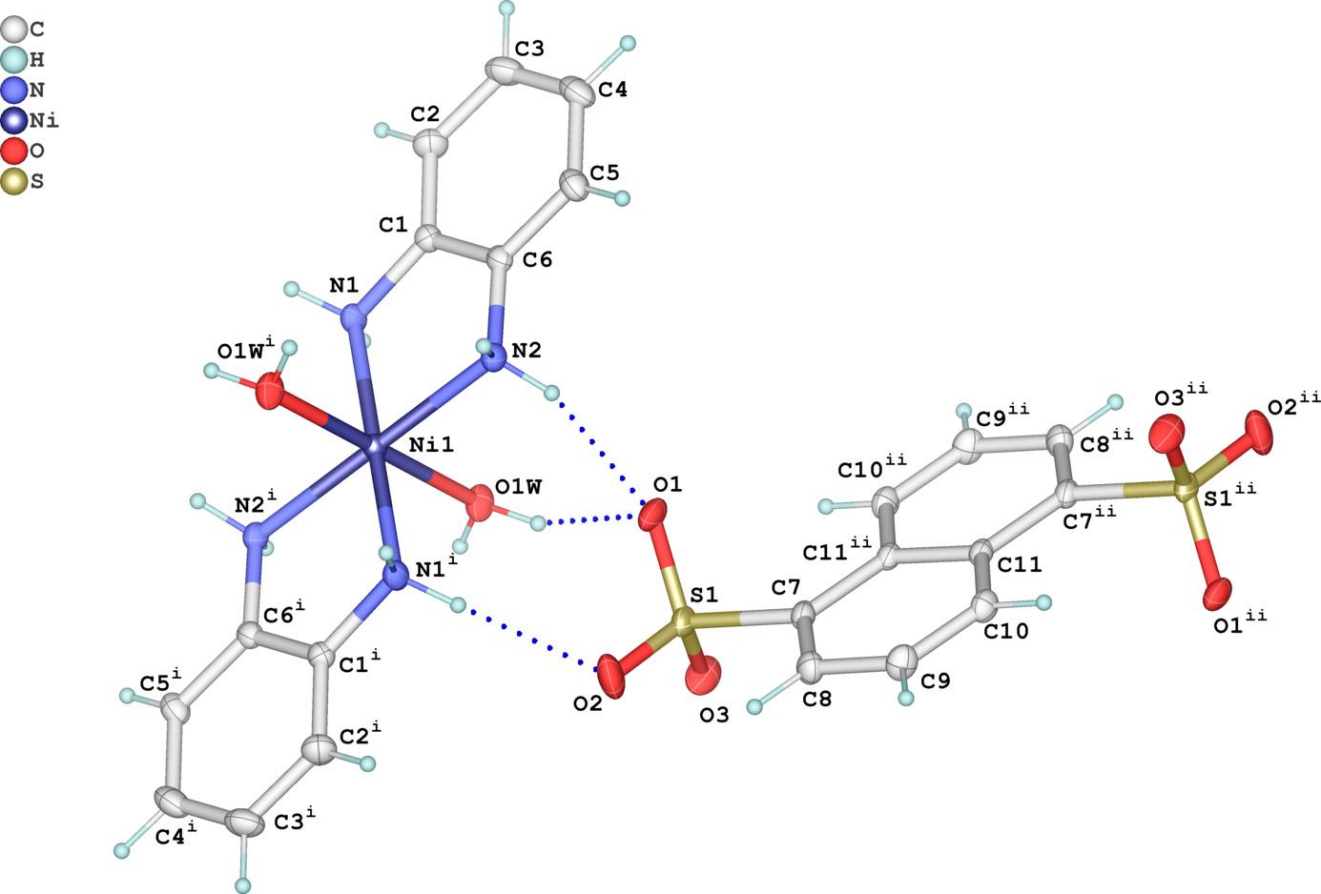
The structures of the mol­ecular entities in the title salt, showing the atom-labeling scheme and displacement ellipsoids drawn at the 50% probability level. H atoms are shown as small spheres of arbitrary radius and hydrogen bonds are shown as dashed lines. [Symmetry codes: (i) 2 − *x*, 1 − *y*, 1 − *z*; (ii) 1 − *x*, 2 − *y*, 1 − *z*.]

The structures of the mol­ecular entities of the title compound are shown in Fig. 1 ▸. This salt consists of an Ni^II^-centered complex cation with two bidentate OPD ligands and *trans*-aligned aqua ligands, as well as of a non-coordinating NDS^2–^ anion, which is the double-deprotonated form of H_2_NDS. The Ni^II^ atom is situated at a crystallographic inversion center (Wyckoff letter *d* of space group *P*2_1_/*n*) and exhibits a slightly tetra­gonally distorted {O_2_N_4_} octa­hedral coordination environment, with two pairs of shorter equatorial Ni—N bonds [2.0775 (17) and 2.0924 (18) Å] and a pair of longer axial Ni—O bonds [2.1381 (17) Å]. The OPD ligand, likewise located over a crystallographic inversion center at the middle of the central C11—C11(−*x* + 1, −*y* + 2, −*z* + 1) bond, is almost coplanar with the NiN_4_ plane, with a dihedral angle of 0.95 (9)°. The deviation of the ideal octa­hedral coordination sphere around nickel might be explained as follows: The inflexible nature of the OPDA ring system with an N⋯N distance between the amino groups of 2.770 (2) Å determines the N2—Ni1—N1 and N2—Ni1—N1(−*x* + 2, −*y* + 1, −*z* + 1) bite angles of 83.26 (7) and 96.74 (7) °, respectively.

## 3. Supra­molecular features and Hirshfeld surface analysis

**Figure 2:**
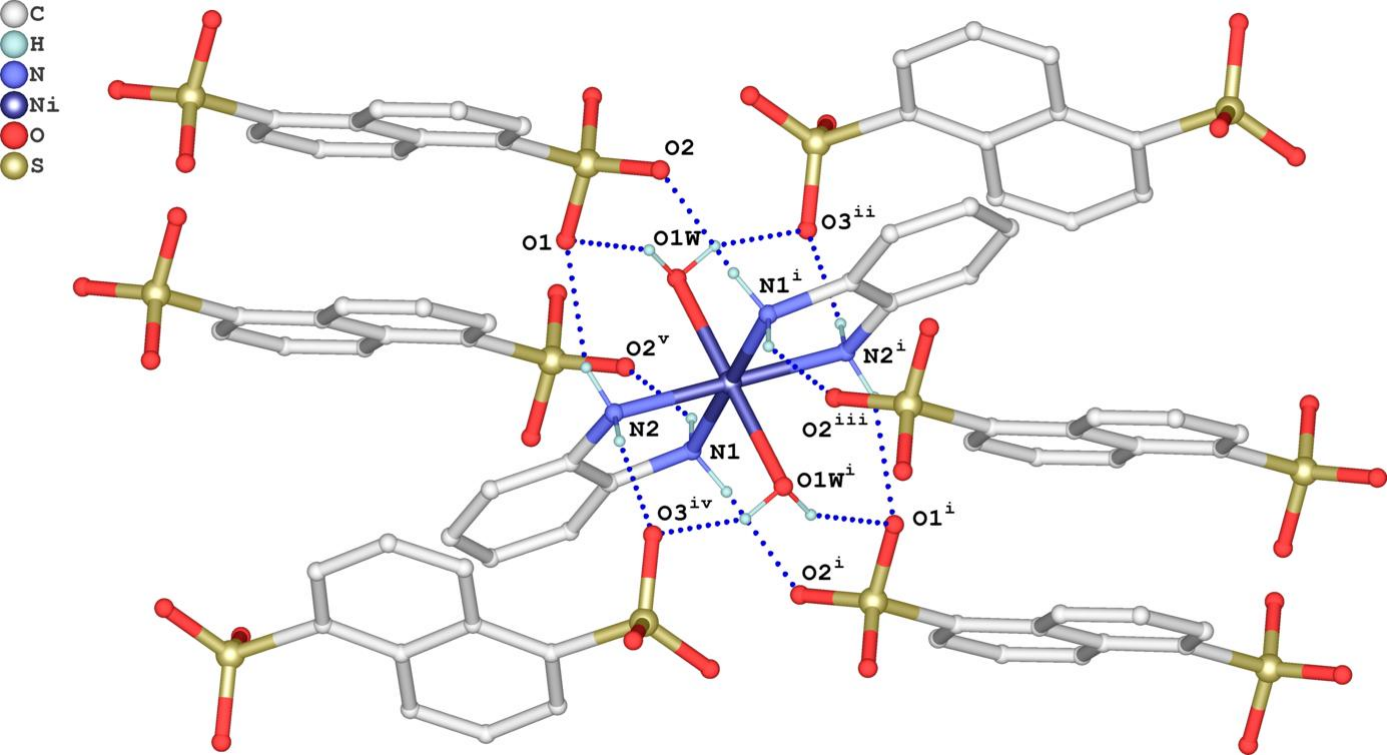
Formation of hydrogen bonds (dashed lines) between the [Ni(OPD)_2_(H_2_O)_2_]^2+^ cation with six neighboring anions. Symmetry codes refer to Table 1 ▸.

**Figure 3:**
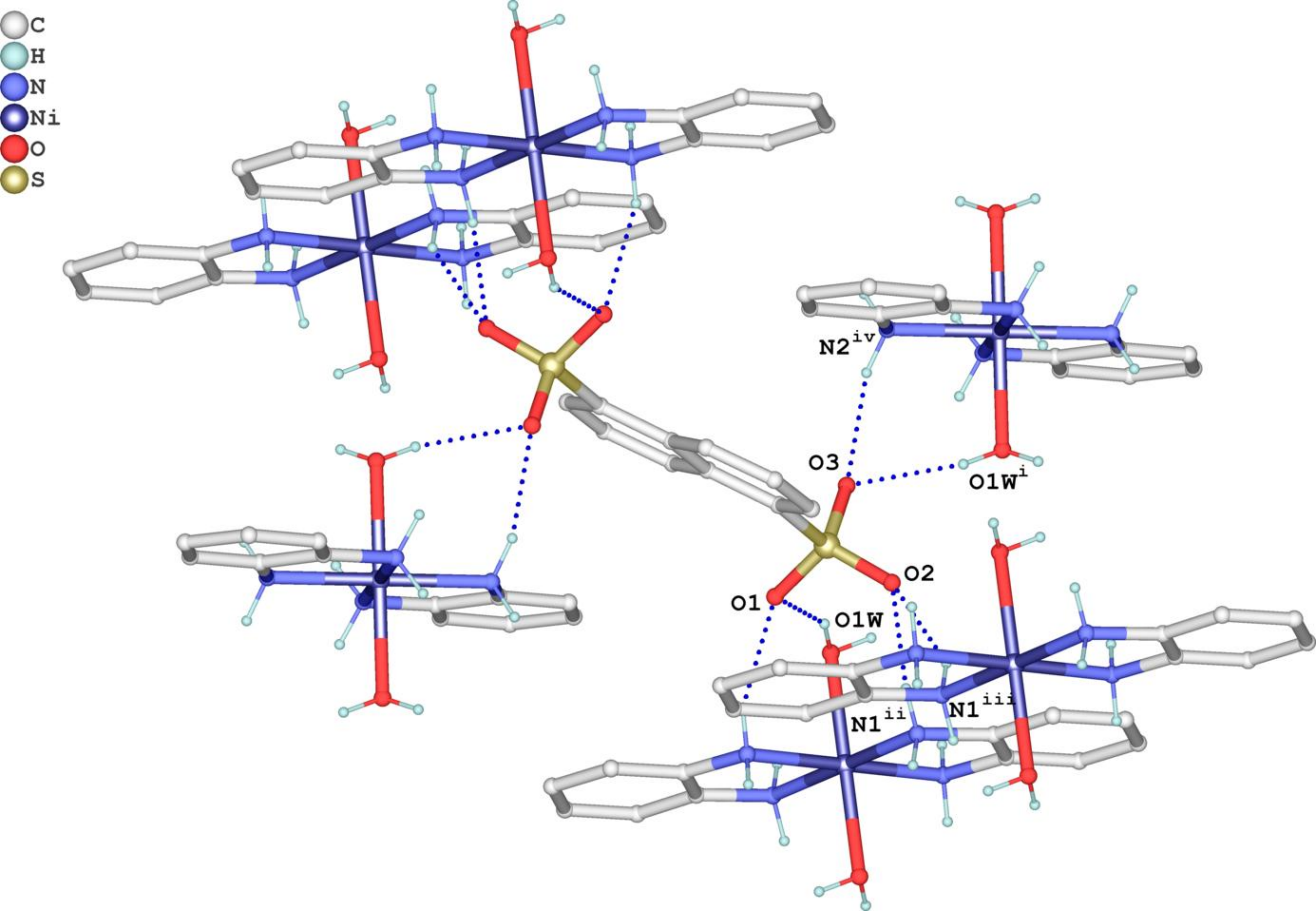
Formation of hydrogen bonds (dashed lines) between the NDS^2–^ anion with six neighboring cations. Symmetry codes refer to Table 1 ▸.

**Figure 4:**
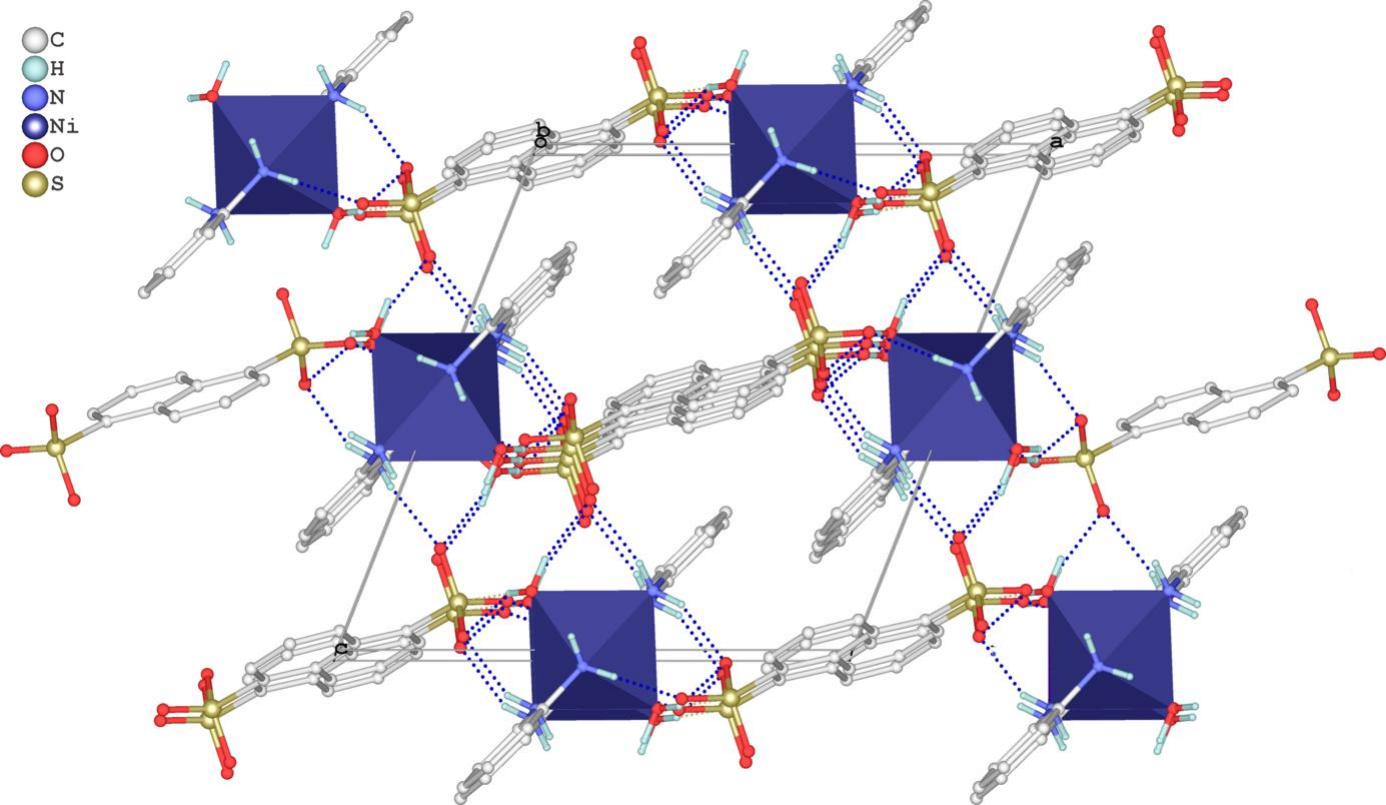
The crystal packing of the title salt in a view along [010] based on the formation of O—H⋯O and N—H⋯O hydrogen bonds (dashed blue lines). The coordination sphere around Ni^II^ is given in the polyhedral representation.

In the crystal, the complex Ni(OPD)_2_(H_2_O)_2_]^2+^ cation and the NDS^2–^ anion are associated *via* charge-assisted inter­molecular O—H⋯O and N—H⋯O hydrogen bonds (Table 1 ▸). Each [Ni(OPD)_2_(H_2_O)_2_]^2+^ cation forms N—H⋯O and O—H⋯O hydrogen bonds with six neighboring organic anions whereby the two aqua and two OPD ligands act solely as hydrogen-bonding donor groups (Fig. 2 ▸). All six acceptor oxygen atoms of the SO_3_^−^ groups of the NDS^2−^ anions participate as double acceptor atoms (Fig. 3 ▸). Hydrogen bonds N1—H1*B*⋯O2^iii^, N2—H2*A*⋯O1 and O1*W*–H1*WB*⋯O1 lead to the formation of supra­molecular zigzag chains parallel to [100]. These chains are further connected by N1—H1*A*⋯O2^ii^ and N1—H1*B*⋯O2^iii^ hydrogen bonds, resulting in sheets parallel to (110). Additionally, cations and neighboring dianions are linked through O1*W*—H1*WA*⋯O3^iv^ and N2—H2*B*⋯O3^i^ hydrogen bonds. The mol­ecules stack along [001], thereby forming a consolidated tri-periodic supra­molecular network (Fig. 4 ▸).

**Figure 5:**
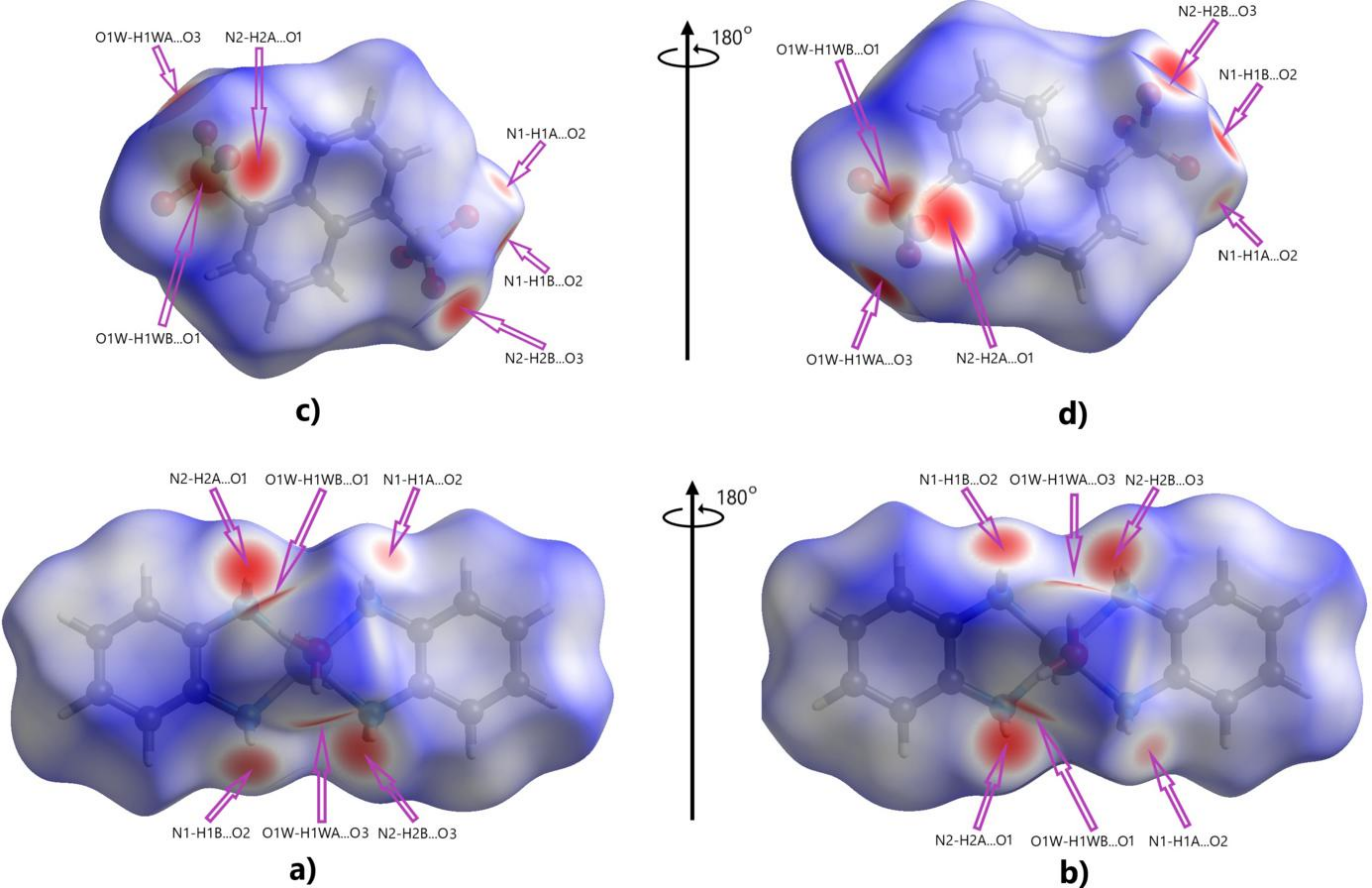
View of the three-dimensional Hirshfeld surface for the [Ni(OPD)_2_(H_2_O)_2_]^2+^ cation and the NDS^2–^ dianion plotted over *d*_norm_. Parts (*a*) and (*b*) show the front and back sides, respectively, of the [Ni(OPD)_2_(H_2_O)_2_]^2+^ dication. Parts (*c*) and (*d*) show the front and back sides, respectively, of the NDS^2–^ dianion.

**Figure 6:**
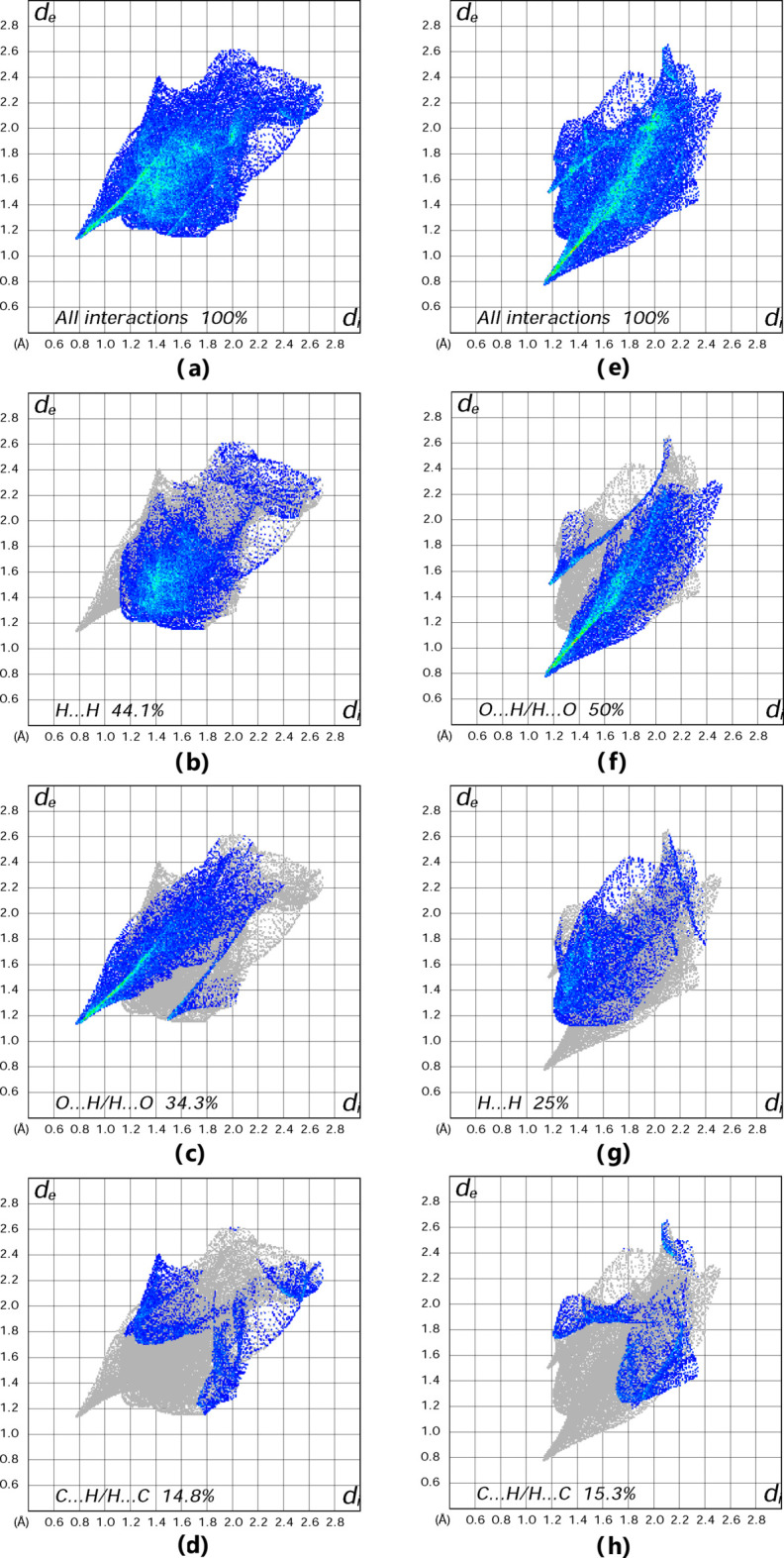
Two-dimensional fingerprint plots for the [Ni(OPD)_2_(H_2_O)_2_]^2+^ cation [parts (*a*), (*b*), (*c*) and (*d*)] and the NDS^2−^dianion [parts (*e*), (*f*), (*g*) and (*h*)]. The *d*_i_ and *d*_e_ values are the closest inter­nal and external distances (in Å) from a given point on the Hirshfeld surface.

The supra­molecular inter­actions discussed above were qu­anti­tatively investigated and visualized using Hirshfeld surface analysis performed with *CrystalExplorer* (Spackman *et al.*, 2021 ▸), with a standard resolution of the three-dimensional *d*_norm_ surfaces plotted over a fixed color scale of −0.5408 (red) to 1.4249 (blue) a.u.. Visualizations were performed using a red–white–blue color scheme, where red highlights contacts shorter than the sum of the van der Waals (vdW) radii, white contacts around vdW separations, and blue contacts longer than the sum of the vdW radii. It should be noted that the Hirshfeld surfaces and fingerprint plots were calculated separately for the [Ni(OPD)_2_(H_2_O)_2_]^2+^ cation and the NDS^2–^ dianion. The *d*_norm_ surface has twelve bright-red spots on the Hirshfeld surface for the cation and anion each (Fig. 5 ▸), resulting from the two O—H⋯O and four N—H⋯O inter­molecular hydrogen bonds, as discussed above (Fig. 5 ▸; the number is doubled due to inversion symmetry for both entities). The classical O—H⋯O and N—H⋯O hydrogen bonds correspond to H⋯H and H⋯O contacts in the two-dimensional fingerprint plots (with contributions of 44.1 and 50% to the Hirshfeld surface for the [Ni(OPD)_2_(H_2_O)_2_]^2+^ cation and NDS^2–^ anion, respectively; Fig. 6 ▸ *b* and 6*f*). O⋯H/H⋯O and C⋯H/H⋯C, inter­actions in the cation, and H⋯H and C⋯H/H⋯C inter­actions in the dianion follow with contributions of 34.3, 14.8, 25 and 15.3%, respectively (Fig. 6 ▸ *c*,*d*,*g*,*h*). Other minor contributions are from C⋯C (6.5%) and C⋯O (0.3%) contacts in the cation, and from C⋯C (8.2%), C⋯O (0.3%), O⋯O (0.1%) and S⋯H (0.1%) contacts in the dianion. The O⋯H/H⋯O contacts are visible as a spike with a sharp tip on the side of the corresponding two-dimensional fingerprint plot, which is indicative of strong inter­molecular inter­actions between atoms. On the other hand, the C⋯H/H⋯C contacts form less pronounced spikes, suggesting that these inter­actions are much weaker.

## 4. Database survey

In a search of the Cambridge Structural Database (CSD, version 2022.3.0; Groom *et al.*, 2016 ▸), a total of 207 compounds containing the *o*-phenyl­enedi­amine moiety were identified. Out of these, 129 compounds were metal complexes, while 78 compounds were organic salts. One organic salt comprising protonated *o*-phenyl­enedi­amine and 1,5-naphthalene­disulfonate has been studied (CSD refcode PEFYOQ; Deng *et al.*, 2012 ▸). When searching with 1,5-naphthalene­disulfonic acid as the search criterion, 90 metal complexes and 170 organic salt compounds were found. In the majority of metal complexes, 1,5-naphthalene­disulfonic acid was found in its dianionic form and was not part of the coordination sphere. However, in ten cases a bridging mode for the 1,5-naphthalene­disulfonate anion was found. Only one compound was identified where the 1,5-naphthalene­disulfonate anion coordinates to a transition-metal cation (copper) in a monodentate manner (XABPEW; Chen *et al.*, 2002 ▸).

## 5. Synthesis and crystallization

The starting materials are commercially available and were used without further purification. The ligand OPDA (0.216 g, 2 mmol) was dissolved in 10 ml of a 1:1 *v*/*v* ethanol/water mixture. This solution was then added to a solution containing nickel(II) sulfate hepta­hydrate (0.281 g, 1 mmol) and disodium naphthalene-1,5-di­sulfonate (0.332 g, 1 mmol) in 10 ml of the same mixed ethanol/water solvent. The resulting mixture was heated under reflux and stirred for 40 min. After 5 d of slow solvent evaporation at room temperature, a light-green crystalline product was obtained with a yield of 65% (based on Ni). Elemental analysis calculated (%) for C_22_H_26_N_4_NiO_8_S_2_: C 44.24, H 4.39, N 9.38; found: C 44.18, H 4.34, N 9.31.

## 6. Refinement

Crystal data, data collection and structure refinement details are summarized in Table 2 ▸. C-bound H atoms were placed in calculated positions and refined using a riding-model approximation, with *U*_iso_(H) = 1.2*U*_eq_(C) and C—H = 0.93 Å for aromatic H atoms. Hydrogen atoms of the amino groups and of the water mol­ecule were located using a difference-Fourier map and refined with bond-length restraints of 0.89 (1) and 0.85 (1) Å, respectively.

## Supplementary Material

Crystal structure: contains datablock(s) I. DOI: 10.1107/S2056989023009350/wm5701sup1.cif

Structure factors: contains datablock(s) I. DOI: 10.1107/S2056989023009350/wm5701Isup2.hkl

CCDC reference: 2303464

Additional supporting information:  crystallographic information; 3D view; checkCIF report

## Figures and Tables

**Table 1:** Hydrogen-bond geometry (Å, °)

*D*—H⋯*A*	*D*—H	H⋯*A*	*D*⋯*A*	*D*—H⋯*A*
N1—H1*A*⋯O2^i^	0.89 (1)	2.48 (2)	3.268 (3)	148 (3)
N1—H1*B*⋯O2^ii^	0.89 (1)	2.18 (1)	3.066 (3)	175 (3)
N2—H2*A*⋯O1	0.88 (1)	2.10 (1)	2.941 (2)	160 (3)
N2—H2*B*⋯O3^iii^	0.89 (1)	2.06 (1)	2.908 (3)	158 (2)
O1*W*—H1*WA*⋯O3^iv^	0.85 (1)	2.12 (2)	2.876 (3)	148 (3)
O1*W*—H1*WB*⋯O1	0.85 (1)	2.04 (2)	2.803 (2)	150 (3)

Symmetry codes: (i) 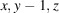 ; (ii) 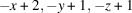 ; (iii) 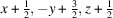 ; (iv) 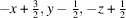 .

**Table 2:** Experimental details

Crystal data	
Chemical formula	[Ni(C_6_H_8_N_2_)_2_(H_2_O)_2_](C_10_H_6_O_6_S_2_)
*M*_r_	597.30
Crystal system, space group	Monoclinic, *P*2_1_/*n*
Temperature (K)	563
*a*, *b*, *c* (Å)	12.7613 (3), 7.7054 (1), 13.4641 (3)
β (°)	111.554 (2)
*V* (Å^3^)	1231.36 (5)
*Z*	2
Radiation type	Cu *K*α
μ (mm^−1^)	3.22
Crystal size (mm)	0.22 × 0.18 × 0.14

Data collection	
Diffractometer	XtaLAB Synergy, Single source at home/near, HyPix3000
Absorption correction	Multi-scan (*CrysAlis PRO*; Rigaku OD, 2023 ▸)
*T*_min_, *T*_max_	0.573, 1.000
No. of measured, independent and observed [*I* > 2σ(*I*)] reflections	11384, 2380, 2152
*R*_int_	0.038
(sin θ/λ)_max_ (Å^−1^)	0.614

Refinement	
*R*[*F* ^2^ > 2σ(*F* ^2^)], *wR*(*F* ^2^), *S*	0.036, 0.097, 1.04
No. of reflections	2380
No. of parameters	194
No. of restraints	6
H-atom treatment	H atoms treated by a mixture of independent and constrained refinement
Δρ_max_, Δρ_min_ (e Å^−3^)	0.50, −0.49

Computer programs: *CrysAlis PRO* (Rigaku OD, 2023 ▸), *SHELXT* (Sheldrick, 2015*a* ▸), *SHELXL* (Sheldrick, 2015*b* ▸), *OLEX2* (Dolomanov *et al.*, 2009 ▸).

## supplementary crystallographic information

### Crystal data

**Table:**

[Ni(C_6_H_8_N_2_)_2_(H_2_O)_2_](C_10_H_6_O_6_S_2_)	*F*(000) = 620
*M**_r_* = 597.30	*D*_x_ = 1.611 Mg m^−^^3^
Monoclinic, *P*2_1_/*n*	Cu *K*α radiation, λ = 1.54184 Å
*a* = 12.7613 (3) Å	Cell parameters from 6737 reflections
*b* = 7.7054 (1) Å	θ = 4.1–71.0°
*c* = 13.4641 (3) Å	µ = 3.22 mm^−^^1^
β = 111.554 (2)°	*T* = 563 K
*V* = 1231.36 (5) Å^3^	Block, light green
*Z* = 2	0.22 × 0.18 × 0.14 mm

### Data collection

**Table:**

XtaLAB Synergy, Single source at home/near, HyPix3000 diffractometer	2380 independent reflections
Radiation source: micro-focus sealed X-ray tube, PhotonJet (Cu) X-ray Source	2152 reflections with *I* > 2σ(*I*)
Mirror monochromator	*R*_int_ = 0.038
Detector resolution: 10.0000 pixels mm^-1^	θ_max_ = 71.3°, θ_min_ = 4.1°
ω scans	*h* = −15→15
Absorption correction: multi-scan (CrysAlisPro; Rigaku OD, 2023)	*k* = −9→9
*T*_min_ = 0.573, *T*_max_ = 1.000	*l* = −14→16
11384 measured reflections	

### Refinement

**Table:**

Refinement on *F*^2^	Hydrogen site location: mixed
Least-squares matrix: full	H atoms treated by a mixture of independent and constrained refinement
*R*[*F*^2^ > 2σ(*F*^2^)] = 0.036	*w* = 1/[σ^2^(*F*_o_^2^) + (0.048*P*)^2^ + 0.8302*P*] where *P* = (*F*_o_^2^ + 2*F*_c_^2^)/3
*wR*(*F*^2^) = 0.097	(Δ/σ)_max_ = 0.001
*S* = 1.04	Δρ_max_ = 0.50 e Å^−^^3^
2380 reflections	Δρ_min_ = −0.49 e Å^−^^3^
194 parameters	Extinction correction: SHELXL (Sheldrick, 2015a), Fc^*^=kFc[1+0.001xFc^2^λ^3^/sin(2θ)]^-1/4^
6 restraints	Extinction coefficient: 0.0006 (2)
Primary atom site location: iterative	

### Special details

**Table:**

Geometry. All esds (except the esd in the dihedral angle between two l.s. planes) are estimated using the full covariance matrix. The cell esds are taken into account individually in the estimation of esds in distances, angles and torsion angles; correlations between esds in cell parameters are only used when they are defined by crystal symmetry. An approximate (isotropic) treatment of cell esds is used for estimating esds involving l.s. planes.

### Fractional atomic coordinates and isotropic or equivalent isotropic displacement parameters (Å^2^)

**Table:**

	*x*	*y*	*z*	*U*_iso_*/*U*_eq_	
Ni1	1.000000	0.500000	0.500000	0.02713 (17)	
S1	0.69836 (5)	0.87424 (8)	0.39745 (4)	0.03779 (18)	
O1W	0.83329 (14)	0.4677 (2)	0.38469 (14)	0.0412 (4)	
O1	0.73435 (15)	0.7254 (2)	0.46852 (14)	0.0488 (5)	
N1	0.99045 (16)	0.2387 (2)	0.53808 (16)	0.0330 (4)	
O3	0.62288 (17)	0.8217 (3)	0.29283 (14)	0.0574 (5)	
N2	0.93181 (15)	0.5408 (2)	0.61643 (14)	0.0298 (4)	
O2	0.79217 (17)	0.9758 (3)	0.39332 (19)	0.0636 (6)	
C11	0.47191 (16)	1.0579 (3)	0.52339 (16)	0.0276 (4)	
C6	0.91414 (17)	0.3765 (3)	0.66003 (16)	0.0297 (4)	
C1	0.94563 (18)	0.2253 (3)	0.62287 (17)	0.0319 (5)	
C7	0.62041 (18)	1.0098 (3)	0.45202 (17)	0.0313 (5)	
C10	0.50918 (19)	1.2316 (3)	0.54324 (18)	0.0350 (5)	
H10	0.473811	1.306841	0.575033	0.042*	
C5	0.86718 (19)	0.3671 (3)	0.73833 (18)	0.0381 (5)	
H5	0.846130	0.468202	0.763862	0.046*	
C8	0.65220 (18)	1.1796 (3)	0.47028 (19)	0.0385 (5)	
H8	0.711068	1.221455	0.451995	0.046*	
C9	0.5960 (2)	1.2909 (3)	0.5166 (2)	0.0405 (5)	
H9	0.618250	1.406318	0.529312	0.049*	
C2	0.9316 (2)	0.0661 (3)	0.6643 (2)	0.0446 (6)	
H2	0.953980	−0.035050	0.639981	0.054*	
C4	0.8518 (2)	0.2078 (4)	0.7781 (2)	0.0474 (6)	
H4	0.819333	0.201834	0.829604	0.057*	
C3	0.8845 (2)	0.0572 (4)	0.7417 (2)	0.0510 (6)	
H3	0.874888	−0.049782	0.769179	0.061*	
H2A	0.8674 (14)	0.596 (3)	0.585 (2)	0.049 (8)*	
H2B	0.9775 (17)	0.607 (3)	0.6689 (15)	0.038 (7)*	
H1A	0.946 (2)	0.188 (4)	0.4786 (15)	0.062 (9)*	
H1B	1.0556 (14)	0.182 (3)	0.561 (2)	0.052 (8)*	
H1WA	0.822 (3)	0.447 (5)	0.3199 (11)	0.072 (11)*	
H1WB	0.787 (2)	0.546 (4)	0.386 (3)	0.085 (12)*	

### Atomic displacement parameters (Å^2^)

**Table:**

	*U*^11^	*U*^22^	*U*^33^	*U*^12^	*U*^13^	*U*^23^
Ni1	0.0287 (3)	0.0260 (3)	0.0306 (3)	0.00340 (18)	0.0155 (2)	0.00141 (19)
S1	0.0365 (3)	0.0456 (3)	0.0367 (3)	0.0168 (2)	0.0198 (2)	0.0050 (2)
O1W	0.0343 (9)	0.0495 (10)	0.0395 (9)	0.0062 (7)	0.0133 (7)	−0.0073 (8)
O1	0.0481 (10)	0.0549 (11)	0.0464 (10)	0.0299 (8)	0.0208 (8)	0.0131 (8)
N1	0.0353 (10)	0.0280 (9)	0.0418 (11)	0.0046 (8)	0.0212 (9)	0.0015 (8)
O3	0.0633 (12)	0.0736 (14)	0.0348 (9)	0.0262 (10)	0.0175 (9)	−0.0035 (9)
N2	0.0291 (9)	0.0302 (9)	0.0316 (9)	0.0041 (7)	0.0131 (8)	−0.0002 (7)
O2	0.0550 (12)	0.0644 (13)	0.0938 (17)	0.0090 (10)	0.0538 (12)	0.0030 (11)
C11	0.0259 (9)	0.0302 (10)	0.0258 (9)	0.0070 (8)	0.0085 (8)	0.0004 (8)
C6	0.0246 (10)	0.0356 (11)	0.0280 (10)	0.0001 (8)	0.0088 (8)	0.0024 (8)
C1	0.0294 (10)	0.0331 (11)	0.0347 (11)	0.0014 (8)	0.0135 (9)	0.0040 (9)
C7	0.0279 (10)	0.0368 (11)	0.0300 (10)	0.0089 (8)	0.0117 (9)	0.0014 (8)
C10	0.0348 (11)	0.0331 (11)	0.0374 (11)	0.0067 (9)	0.0137 (9)	−0.0044 (9)
C5	0.0349 (11)	0.0492 (14)	0.0331 (11)	−0.0016 (10)	0.0158 (9)	−0.0019 (10)
C8	0.0286 (11)	0.0411 (12)	0.0475 (13)	0.0015 (9)	0.0160 (10)	0.0034 (10)
C9	0.0386 (12)	0.0301 (11)	0.0516 (14)	−0.0005 (9)	0.0153 (11)	−0.0049 (10)
C2	0.0493 (14)	0.0354 (12)	0.0517 (15)	0.0012 (11)	0.0217 (12)	0.0085 (11)
C4	0.0461 (14)	0.0645 (17)	0.0366 (12)	−0.0081 (12)	0.0209 (11)	0.0059 (12)
C3	0.0573 (16)	0.0501 (15)	0.0487 (15)	−0.0050 (13)	0.0231 (13)	0.0153 (12)

### Geometric parameters (Å, º)

**Table:**

Ni1—O1W^i^	2.1381 (17)	C11—C7^ii^	1.434 (3)
Ni1—O1W	2.1381 (17)	C11—C10	1.413 (3)
Ni1—N1	2.0924 (18)	C6—C1	1.385 (3)
Ni1—N1^i^	2.0924 (18)	C6—C5	1.393 (3)
Ni1—N2^i^	2.0776 (17)	C1—C2	1.386 (3)
Ni1—N2	2.0775 (17)	C7—C8	1.365 (3)
S1—O1	1.4558 (18)	C10—H10	0.9300
S1—O3	1.4419 (19)	C10—C9	1.363 (3)
S1—O2	1.448 (2)	C5—H5	0.9300
S1—C7	1.777 (2)	C5—C4	1.382 (4)
O1W—H1WA	0.846 (10)	C8—H8	0.9300
O1W—H1WB	0.850 (10)	C8—C9	1.403 (3)
N1—C1	1.456 (3)	C9—H9	0.9300
N1—H1A	0.886 (10)	C2—H2	0.9300
N1—H1B	0.889 (10)	C2—C3	1.383 (4)
N2—C6	1.447 (3)	C4—H4	0.9300
N2—H2A	0.882 (10)	C4—C3	1.383 (4)
N2—H2B	0.891 (10)	C3—H3	0.9300
C11—C11^ii^	1.427 (4)		
			
O1W—Ni1—O1W^i^	180.0	H2A—N2—H2B	109 (3)
N1^i^—Ni1—O1W^i^	86.20 (8)	C11^ii^—C11—C7^ii^	117.6 (2)
N1^i^—Ni1—O1W	93.80 (8)	C10—C11—C11^ii^	119.1 (2)
N1—Ni1—O1W^i^	93.80 (8)	C10—C11—C7^ii^	123.28 (19)
N1—Ni1—O1W	86.20 (8)	C1—C6—N2	118.72 (18)
N1—Ni1—N1^i^	180.0	C1—C6—C5	119.5 (2)
N2^i^—Ni1—O1W	90.88 (7)	C5—C6—N2	121.8 (2)
N2^i^—Ni1—O1W^i^	89.12 (7)	C6—C1—N1	118.23 (19)
N2—Ni1—O1W	89.12 (7)	C6—C1—C2	120.1 (2)
N2—Ni1—O1W^i^	90.88 (7)	C2—C1—N1	121.6 (2)
N2^i^—Ni1—N1^i^	83.26 (7)	C11^ii^—C7—S1	120.95 (16)
N2—Ni1—N1^i^	96.74 (7)	C8—C7—S1	117.60 (17)
N2—Ni1—N1	83.26 (7)	C8—C7—C11^ii^	121.45 (19)
N2^i^—Ni1—N1	96.74 (7)	C11—C10—H10	119.4
N2—Ni1—N2^i^	180.00 (10)	C9—C10—C11	121.2 (2)
O1—S1—C7	106.30 (10)	C9—C10—H10	119.4
O3—S1—O1	110.91 (13)	C6—C5—H5	119.9
O3—S1—O2	112.29 (13)	C4—C5—C6	120.1 (2)
O3—S1—C7	107.13 (10)	C4—C5—H5	119.9
O2—S1—O1	112.67 (12)	C7—C8—H8	120.0
O2—S1—C7	107.12 (11)	C7—C8—C9	120.0 (2)
Ni1—O1W—H1WA	121 (2)	C9—C8—H8	120.0
Ni1—O1W—H1WB	115 (3)	C10—C9—C8	120.7 (2)
H1WA—O1W—H1WB	107 (3)	C10—C9—H9	119.7
Ni1—N1—H1A	106 (2)	C8—C9—H9	119.7
Ni1—N1—H1B	115 (2)	C1—C2—H2	119.9
C1—N1—Ni1	109.60 (13)	C3—C2—C1	120.2 (2)
C1—N1—H1A	112 (2)	C3—C2—H2	119.9
C1—N1—H1B	106.1 (19)	C5—C4—H4	119.9
H1A—N1—H1B	108 (3)	C5—C4—C3	120.2 (2)
Ni1—N2—H2A	107.0 (18)	C3—C4—H4	119.9
Ni1—N2—H2B	110.6 (16)	C2—C3—H3	120.1
C6—N2—Ni1	110.14 (13)	C4—C3—C2	119.8 (2)
C6—N2—H2A	110.8 (19)	C4—C3—H3	120.1
C6—N2—H2B	109.3 (17)		
			
Ni1—N1—C1—C6	2.3 (2)	O2—S1—C7—C8	5.8 (2)
Ni1—N1—C1—C2	−179.29 (18)	C11^ii^—C11—C10—C9	1.3 (4)
Ni1—N2—C6—C1	1.6 (2)	C11^ii^—C7—C8—C9	1.7 (3)
Ni1—N2—C6—C5	−178.80 (17)	C11—C10—C9—C8	−1.1 (4)
S1—C7—C8—C9	−177.84 (18)	C6—C1—C2—C3	1.0 (4)
O1—S1—C7—C11^ii^	−53.0 (2)	C6—C5—C4—C3	1.0 (4)
O1—S1—C7—C8	126.48 (19)	C1—C6—C5—C4	−0.3 (3)
N1—C1—C2—C3	−177.3 (2)	C1—C2—C3—C4	−0.3 (4)
O3—S1—C7—C11^ii^	65.6 (2)	C7^ii^—C11—C10—C9	−178.7 (2)
O3—S1—C7—C8	−114.9 (2)	C7—C8—C9—C10	−0.4 (4)
N2—C6—C1—N1	−2.7 (3)	C5—C6—C1—N1	177.67 (19)
N2—C6—C1—C2	178.9 (2)	C5—C6—C1—C2	−0.8 (3)
N2—C6—C5—C4	−179.9 (2)	C5—C4—C3—C2	−0.7 (4)
O2—S1—C7—C11^ii^	−173.73 (18)		

Symmetry codes: (i) −*x*+2, −*y*+1, −*z*+1; (ii) −*x*+1, −*y*+2, −*z*+1.

### Hydrogen-bond geometry (Å, º)

**Table:**

*D*—H···*A*	*D*—H	H···*A*	*D*···*A*	*D*—H···*A*
N1—H1*A*···O2^iii^	0.89 (1)	2.48 (2)	3.268 (3)	148 (3)
N1—H1*B*···O2^i^	0.89 (1)	2.18 (1)	3.066 (3)	175 (3)
N2—H2*A*···O1	0.88 (1)	2.10 (1)	2.941 (2)	160 (3)
N2—H2*B*···O3^iv^	0.89 (1)	2.06 (1)	2.908 (3)	158 (2)
O1*W*—H1*WA*···O3^v^	0.85 (1)	2.12 (2)	2.876 (3)	148 (3)
O1*W*—H1*WB*···O1	0.85 (1)	2.04 (2)	2.803 (2)	150 (3)

Symmetry codes: (i) −*x*+2, −*y*+1, −*z*+1; (iii) *x*, *y*−1, *z*; (iv) *x*+1/2, −*y*+3/2, *z*+1/2; (v) −*x*+3/2, *y*−1/2, −*z*+1/2.

## References

[bb1] Abdullah, Kh., Hussien, N., Salam, A., Kareem, A., Rahman, A. & Mourad, S. (2019). *Biochem. Cell. Arch.* **19**, 1705–1711.

[bb2] Adhikari, S., Bhattacharjee, T., Bhattacharjee, S., Daniliuc, C. G., Frontera, A., Lopato, E. M. & Bernhard, S. (2021). *Dalton Trans.* **50**, 5632–5643. 10.1039/d1dt00352f 33908954

[bb3] Aniket, P., Shantanu, D. S., Anagha, O. B. & Ajinkya, P. S. (2015). *Int. J. ChemTech Res.* **8**, 496–500.

[bb4] Bottari, F., Moro, G., Sleegers, N., Florea, A., Cowen, T., Piletsky, S., van Nuijs, A. L. N. & De Wael, K. (2020). *Electroanalysis*, **32**, 135–141.

[bb5] Chakraborty, P., Jana, A. & Mohanta, S. (2014). *Polyhedron*, **77**, 39–46.

[bb6] Chen, B., Ye, W., Li, Z., Jin, S., Wang, J., Guo, M. & Wang, D. (2022). *J. Mol. Struct.* **1249**, 131602.

[bb7] Chen, C. H., Cai, J. W., Feng, X. L. & Chen, X. M. (2002). *Chin. J. Inorg. Chem.* **18**, 659–664.

[bb8] Chen, J., Li, J., Fu, X., Xie, Q., Zeng, T., Jin, S., Xu, W. & Wang, D. (2020). *J. Mol. Struct.* **1204**, 127491.

[bb9] Das, D., Mahata, G., Adhikary, A., Konar, S. & Biradha, K. (2015). *Cryst. Growth Des.* **15**, 4132–4141.

[bb10] Deng, Z.-P., Huo, L.-H., Zhao, H. & Gao, S. (2012). *Cryst. Growth Des.* **12**, 3342–3355.

[bb11] Djebli, Y., Boufas, S., Bencharif, L., Roisnel, T. & Bencharif, M. (2012). *Acta Cryst.* E**68**, m1411–m1412. 10.1107/S1600536812041967 PMC3515150 23284377

[bb12] Dolomanov, O. V., Bourhis, L. J., Gildea, R. J., Howard, J. A. K. & Puschmann, H. (2009). *J. Appl. Cryst.* **42**, 339–341.

[bb13] González Guillén, A., Oszajca, M., Luberda-Durnaś, K., Gryl, M., Bartkiewicz, S., Miniewicz, A. & Lasocha, W. (2018). *Cryst. Growth Des.* **18**, 5029–5037.

[bb14] Groom, C. R., Bruno, I. J., Lightfoot, M. P. & Ward, S. C. (2016). *Acta Cryst.* B**72**, 171–179. 10.1107/S2052520616003954 PMC4822653 27048719

[bb15] Huo, L.-H., Gao, S., Xu, S.-X. & Zhao, H. (2005). *Acta Cryst.* E**61**, m449–m450.

[bb16] Jadoun, S., Riaz, U., Yáñez, J. & Pal Singh Chauhan, N. (2021). *Eur. Polym. J.* **156**, 110600.

[bb17] Kokunov, Yu. V., Kovalev, V. V., Gorbunova, Yu. E. & Kozyukhin, S. A. (2015). *Russ. J. Inorg. Chem.* **60**, 151–156.

[bb18] Konieczny, P., González-Guillén, A. B., Luberda-Durnaś, K., Čižmár, E., Pełka, R., Oszajca, M. & Łasocha, W. (2019). *Dalton Trans.* **48**, 7560–7570. 10.1039/c9dt00624a 30941384

[bb19] Koroteev, P. S., Ilyukhin, A. B., Babeshkin, K. A. & Efimov, N. N. (2020). *J. Mol. Struct.* **1207**, 127800.

[bb20] Lian, Z. & Qu, J. (2013). *Z. Kristallogr. New Cryst. Struct.* **228**, 482–484.

[bb21] Lu, X., Wang, Z. & Liu, M. (2009). *Chin. J. Chem.* **27**, 221–226.

[bb22] Ngopoh, F. A. I., Lachkar, M., Đorđević, T., Lengauer, C. L. & El Bali, B. (2015). *J. Chem. Crystallogr.* **45**, 369–375.

[bb23] Oh, H., Kim, D., Kim, D., Park, I.-H. & Jung, O.-S. (2020). *Cryst. Growth Des.* **20**, 7027–7033.

[bb24] Pardeshi, S. D. & Thore, S. N. (2015). *Int. J. Chem. Phys.* **4**, 300–307.

[bb25] Pisarevskaya, E. Y., Kolesnichenko, I. I., Averin, A. A., Gorbunov, A. M. & Efimov, O. N. (2020). *Synth. Met.* **270**, 116596.

[bb26] Rigaku OD (2023). *CrysAlis PRO*. Rigaku Oxford Diffraction Ltd, Yarnton, Oxfordshire, England.

[bb27] Sabbani, S. & Das, S. K. (2009). *Inorg. Chem. Commun.* **12**, 364–367.

[bb28] Sagasser, J., Ma, B. N., Baecker, D., Salcher, S., Hermann, M., Lamprecht, J., Angerer, S., Obexer, P., Kircher, B. & Gust, R. (2019). *J. Med. Chem.* **62**, 8053–8061. 10.1021/acs.jmedchem.9b00814 31369259

[bb29] Sayyah, S. M., El–Deeb, M. M., Kamal, S. M. & Azooz, R. (2009). *J. Appl. Polym. Sci.* **112**, 3695–3706.

[bb30] Sheldrick, G. M. (2015*a*). *Acta Cryst.* A**71**, 3–8.

[bb31] Sheldrick, G. M. (2015*b*). *Acta Cryst.* C**71**, 3–8.

[bb32] Shi, Ch., Wei, B. & Zhang, W. (2014). *Cryst. Growth Des.* **14**, 6570–6580.

[bb33] Spackman, P. R., Turner, M. J., McKinnon, J. J., Wolff, S. K., Grimwood, D. J., Jayatilaka, D. & Spackman, M. A. (2021). *J. Appl. Cryst.* **54**, 1006–1011. 10.1107/S1600576721002910 PMC8202033 34188619

[bb34] Tai, X.-S., Zhang, Y.-P. & Zhao, W.-H. (2015). *Res. Chem. Intermed.* **41**, 4339–4347.

[bb35] Vishvanath, D. P. & Ketan, P. P. (2014). *Int. J. ChemTech Res.* **8**, 457–465.

[bb36] Willett, R. D., Gómez-García, C. J., Twamley, B., Gómez-Coca, S. & Ruiz, E. (2012). *Inorg. Chem.* **51**, 5487–5493. 10.1021/ic3007124 22512477

[bb37] Xu, W., Lu, Y., Xia, Y. Y., Liu, B., Jin, S., Zhong, B., Wang, D. & Guo, M. (2019). *J. Mol. Struct.* **1189**, 81–93.

[bb38] Zick, P. L. & Geiger, D. K. (2016). *Acta Cryst.* E**72**, 1037–1042. 10.1107/S2056989016010033 PMC4992933 27555958

